# Cardiac pacemaker–related endocarditis complicated with pulmonary embolism: Case report

**DOI:** 10.3389/fcvm.2023.1191194

**Published:** 2023-06-16

**Authors:** Dalila Šačić, Olga Petrović, Danijela Zamaklar-Trifunović, Branislava Ivanović

**Affiliations:** ^1^ Faculty of Medicine, University of Belgrade, Belgrade, Serbia; ^2^ Cardiology Clinic, University Clinical Center of Serbia, Belgrade, Serbia

**Keywords:** pacemaker, vegetation, endocarditis, *Staphylococcus*, embolism

## Abstract

Cardiac device–related endocarditis as a device-therapy complication is a growing problem due to higher life expectancy and the increasing number of abandoned leads and subclinical symptoms. We reported a case of a 47-year-old woman with an implanted pacemaker who was admitted to the clinic for cardiology due to the right-sided device-related infective endocarditis of the pacemaker leads with vegetations, predominantly in the right atrium and right ventricle and complicated by pulmonary embolism. Several years after pacemaker implantation, she was diagnosed with systemic lupus erythematosus and started immunosuppressive therapy. The patient was treated with prolonged intravenous antibiotic therapy. The atrial and ventricular lead was extirpated, and the posterior leaflet of the tricuspid valve was shaved.

## 1. Introduction

Cardiac device–related endocarditis has emerged as a serious device-therapy complication in the era of advanced medical technology and is a growing problem due to higher life expectancy, limited electrode lifetime, and an increasing number of abandoned leads and subclinical symptoms (1). We present a case of a pacemaker lead endocarditis complicated by pulmonary embolism in a female patient on immunosuppressive treatment for systemic lupus erythematosus (SLE).

## 2. Case description

A 47-year-old female was admitted to the cardiology clinic with a diagnosis of right-sided device-related infective endocarditis of the pacemaker leads with vegetations, predominantly in the right atrium and right ventricle. The patient's medical history revealed pacemaker implantation (DDDR pacemaker system RV and RA electrode St. Jude Medical 1688 T) due to a complete atrioventricular block. After 10 years, the pacemaker's elective replacement indicator mode was detected, indicating the need for a pacemaker replacement. Post-surgery, the previously implanted ventricular lead could not be disconnected from the generator and was cut and isolated as inactive. A new ventricular lead was implanted in the septal position, and the pulse generator was replaced with new DDDR mode stimulation.

The patient had a history of hypertension, diabetes, and paroxysmal atrial fibrillation prior to hospital admission. She was diagnosed with SLE 9 years after pacemaker implantation and started immunosuppressive therapy with prednisone and chloroquine.

Prior to hospital admission, the patient underwent a dental procedure, following which she exhibited indications of soft tissue inflammation within the oral cavity. Upon admission to the hospital, the patient had a fever of up to 38.3°C and was diagnosed with inflammatory syndrome based on laboratory tests that revealed an erythrocyte sedimentation rate (ESR) of 58, C-reactive protein (CRP) of 148 mg/L, and leukocytes of 16 × 10^9^/L. Blood cultures drawn prior to starting antibiotics grew *Staphylococcus epidermidis*, and antibiotic therapy was administered accordingly (vancomycin 2 g  × 1 g intravenous, gentamicin 2 mg  × 80 mg, rifampicin 600 mg with antifungal fluconazole 150 mg once a week, and a probiotic). The patient tolerated the antibiotic course and received treatment for 6 weeks.

**Figure 1:**
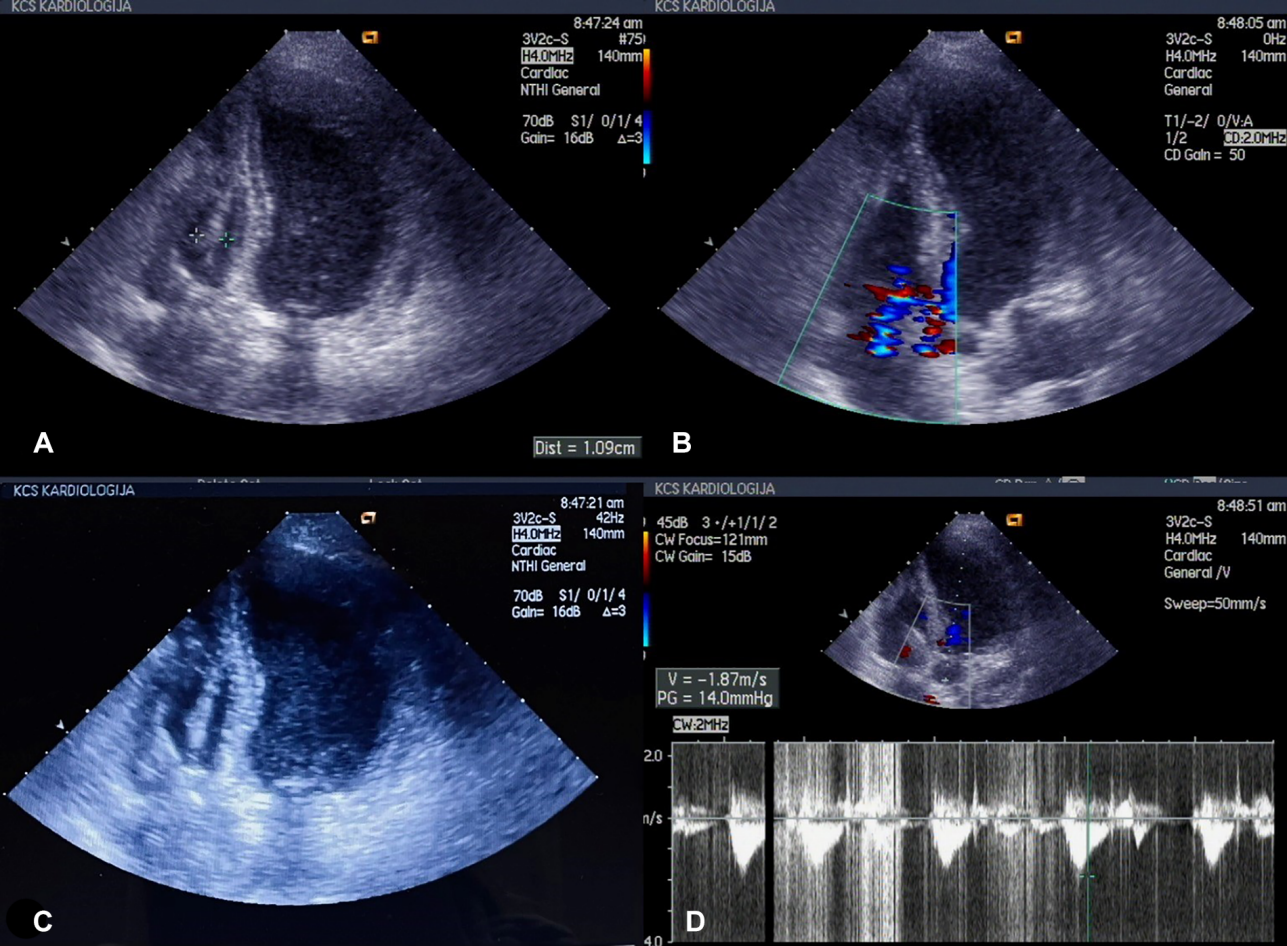
(**A**–**D**) Echonosonography showing vegetations covering the pacemaker electrode in the right ventricle.

Transesophageal echocardiography (TEE) confirmed the diagnosis of right-side lead-associated endocarditis (LAE), with the presence of vegetation on the pacemaker leads, predominantly in the right atrium (maximum of 3 cm) and right ventricle (maximum of 1 cm). Tricuspid valve regurgitation of 2–3 and a small pericardial effusion were also observed (Figure 1).

**Figure 2:**
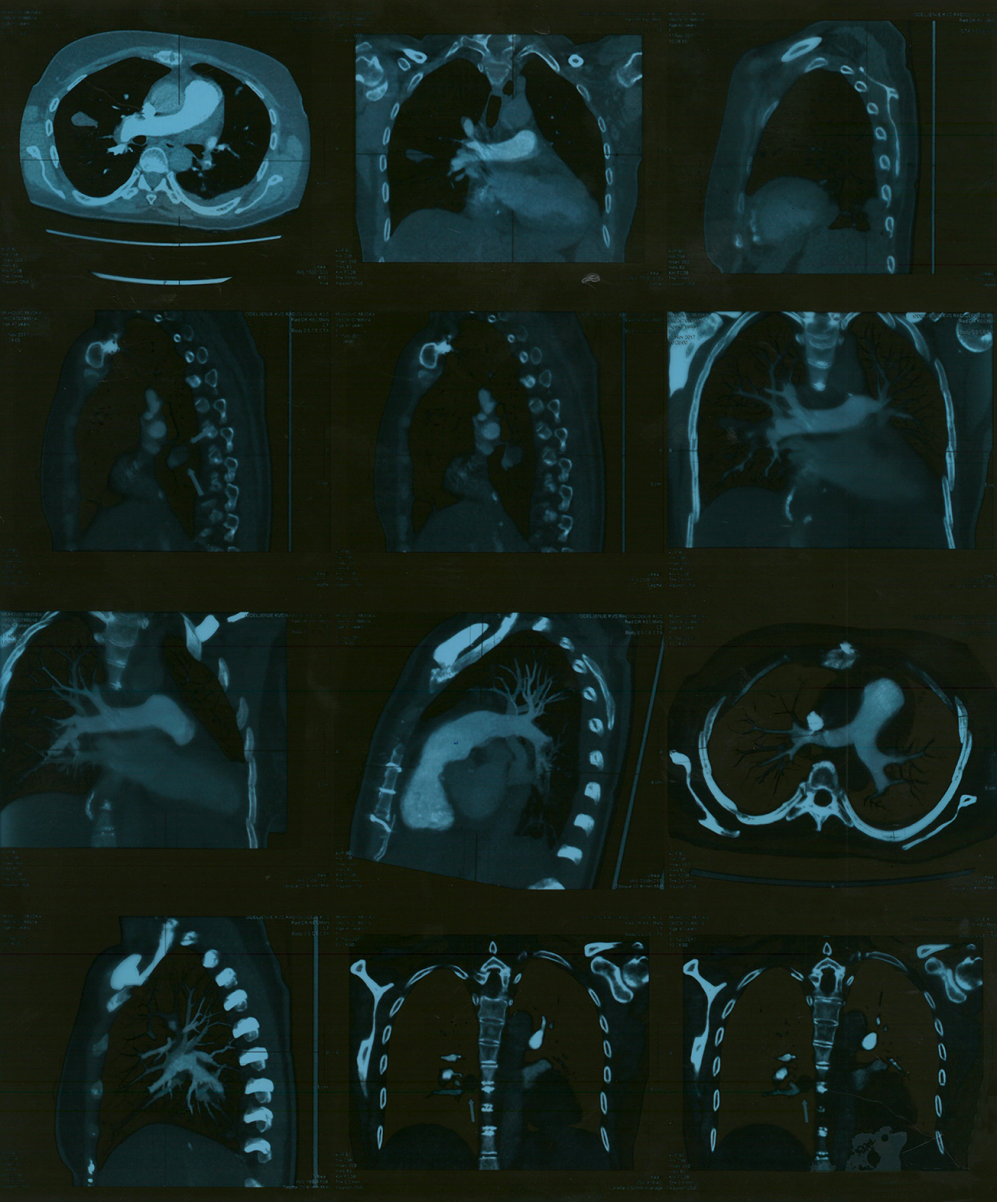
MSCT of coronary blood vessels with massive thromboembolism at the bifurcation of the right pulmonary artery. MSCT, multi-slice CT.

Multi-slice CT (MSCT) coronarography revealed a massive thromboembolism at the bifurcation of the right pulmonary artery, with surrounding muscle or thromboembolism in the left lower segmental branch (Figure 2). Surgical extraction was performed, specifically the extirpation of the atrial and ventricular leads and shaving of the posterior leaf of the tricuspid valve. The patient received prolonged intravenous antibiotics during the treatment.

**Figure 3:**
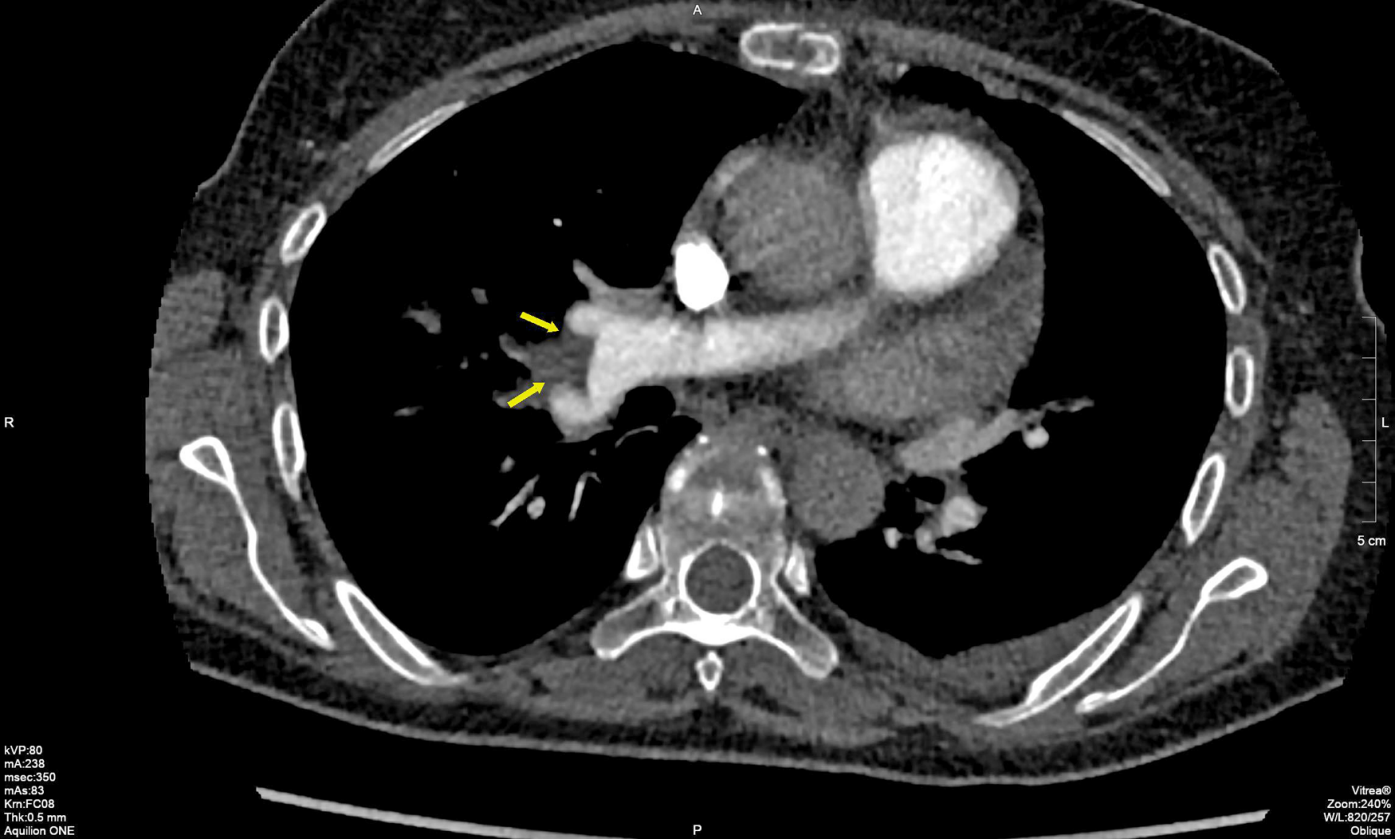
MSCT of the thorax showing small residual thrombotic masses. MSCT, multi-slice CT.

After surgery, the patient was afebrile. Her cardiac action was of a sinus rhythm with a frequency of 66 bpm, and the need for pacemaker reimplantation was ruled out. Blood cultures taken during the continuation of antibiotic treatment and after the completion of treatment were sterile, and laboratory analyses were within normal ranges. A follow-up echocardiographic examination a few days later revealed normal findings, and MSCT of the thorax showed a small amount of left-sided pleural effusion and no pulmonary consolidation or infiltration. A small thrombotic mass (8 mm × 6 mm) was present in the lower lobar branch of the right pulmonary artery and in the branch of the lower-left lobe (8 mm  × 7 mm) (Figure 3). One month after surgery, a control MSCT of the thoracic showed the pulmonary artery without endoluminal pathological masses in the pulmonary lobar and segmental branches.

Written informed consent was obtained from the individual for the publication of any potentially identifiable images or data included in this article.

## 3. Discussion

The rate of cardiac implantable electronic device (CIED) infection, including permanent pacemaker and implantable cardioverter defibrillators, has increased over the past three decades (2–4) due to an aging population of CIED recipients with associated comorbidities, such as diabetes, heart, and renal failure (5). The patient, presenting with infective endocarditis as her primary diagnosis, had also the following comorbidities: diabetes, arterial hypertension, paroxysmal atrial fibrillation, lupus erythematosus systemic, and osteopenia.

Pacemaker infection may be present as either local pocket infection or bloodstream infection with or without LAE or as late-onset lead endocarditis (5, 6). When it comes to the symptoms and signs, fever is the most common symptom. Increased ESR, CRP, leukocytosis, microscopic hematuria, and anemia were the most common laboratory findings (5–9). Our patient also exhibited remarkably similar findings, including elevated levels of CRP, leukocytosis, and ESR.

In addition, this case was specific due to the inactive implanted ventricular lead that was not disconnected from the generator, so it was cut and isolated. A study that investigated the clinical failure-related events and complications of lead dislodgement showed that more deaths were reported from interventions than those from lead-related trauma or embolization (10). This indicates the importance of critical and timely decision-making on the intervention to be carried out.

Staphylococcal species are the predominant organisms responsible for pacemaker infection (9, 11, 12). However, more indolent organisms such as the coagulase-negative staphylococci are important pathogens, particularly in late-onset LAE as was a case in our patient where *Staphylococcus epidermidis* was detected (5, 13).

TEE and computed tomographic angiography (CTA) are currently the first-line imaging studies for device-related endocarditis. A fluorodeoxyglucose positron emission tomography (FDG-PET) scan can detect the site of infection earlier than anatomic findings on TEE and CTA and even earlier than surgical exploration, which is significant for clinical management (14).

Among patients with autoimmune diseases, such as in our case where the patient underwent a dental procedure, infective endocarditis can arise due to pathogen exposure during dental or surgical interventions. It is noteworthy that corticosteroids have been shown to substantially elevate the risk of cardiovascular diseases in patients with SLE, as demonstrated in a study (15).

Studies have shown that infections with coagulase-negative staphylococci are more often associated with larger vegetations (>1 cm) than those in infections with *Staphylococcus aureus*, which more often causes smaller vegetations (<1 cm) (5). This was true in the reported case where a significant amount of pacemaker lead was observed covering the structures (maximum of 3 cm).

Treatment of pacemaker infection includes prompt removal of the device and a prolonged course of intravenous antibiotics (9). There is some concern about performing percutaneous extraction in patients with large lead vegetations due to the risk of pulmonary emboli.

The American Heart Association and Heart Rhythm Society guidelines recommend complete device removal with a prolonged course of antibiotic therapy lasting up to 6 weeks in any patient with an infection (16). Recent European guidelines have emphasized the association of vegetation size with embolic risk in endocarditis (17).

Septic pulmonary embolism may occur in a significant number of patients with LAE, ranging from 31.2% (18) to 55% of them. It was previously observed that the size of the vegetation >15 mm in diameter is a very important predictor of pulmonary embolic events (19). In accordance with the European and American recommendations for our patient, a surgical extraction was performed, i.e., the extirpation of the atrial and ventricular lead. The adequacy of this treatment was validated by Greenspon et al., who concluded that patients who underwent device removal exhibited superior outcomes compared to those who received drug therapy alone (5).

## 4. Conclusion

This case illustrates the complexity of diagnosing, investigating, and managing patients with cardiac device–associated infection that remains a rare but potentially lethal complication of device implantation.

Prompt recognition and management of LAE depend on obtaining blood cultures and echocardiography, including TEE, in all patients who present with either signs of local pocket or systemic infection.

This case underscores the role of prolonged immunosuppression, integral to the management of autoimmune disorders like SLE, as a contributory factor augmenting the susceptibility of patients with pacemaker (PM) to the development of infective endocarditis (IE). This risk is further compounded by well-recognized predisposing factors, such as dental interventions.

The consideration of septic pulmonary embolism should be prioritized in patients with LAE, particularly in cases where the size of the vegetation exceeds 15 mm, as demonstrated in our patient.

## Data Availability

The original contributions presented in the study are included in the article, further inquiries can be directed to the corresponding author.
